# Preliminary Evaluation of Zanubrutinib-Containing Regimens in DLBCL and the Cerebrospinal Fluid Distribution of Zanubrutinib: A 13-Case Series

**DOI:** 10.3389/fonc.2021.760405

**Published:** 2021-12-24

**Authors:** Yan Zhang, Yanan Li, Zhe Zhuang, Wei Wang, Chong Wei, Danqing Zhao, Daobin Zhou, Wei Zhang

**Affiliations:** Department of Hematology, Peking Union Medical College Hospital, Chinese Academy of Medical Sciences (CAMS) & Peking Union Medical College, Beijing, China

**Keywords:** Zanubrutinib, diffuse large B-cell lymphoma, liquid chromatography–tandem mass spectrometry, cerebrospinal fluid, distribution

## Abstract

Zanubrutinib is a second-generation Bruton’s tyrosine kinase inhibitor. Its safety and effectiveness in central nervous system (CNS) lymphoma along with its distribution in the brain and ability to cross the blood–brain barrier (BBB) remain unknown. This retrospective case series involved patients with diffuse large B-cell lymphoma (DLBCL) treated with zanubrutinib-containing regimens from August to December 2020 in PUMCH. The amounts of zanubrutinib in the plasma and brain were assessed by liquid chromatography–tandem mass spectrometry in paired plasma and cerebrospinal fluid (CSF) samples. In total, 13 patients were included: eight primary CNS lymphoma cases and five systemic DLBCL cases with 61.5% (8/13) refractory/relapsed and 84.6% (11/13) showing CNS involvement. The overall response rates were 84.5% in the entire population and 81.8% in the CNS-involved cases. A total of 23 time-matched plasma-CSF sample pairs were collected. The mean peak concentration of zanubrutinib in CSF was 2941.1 pg/ml (range, 466–9032.0 pg/ml). The corrected mean CSF/plasma ratio determined based on 94% protein binding was 42.7% ± 27.7% (range, 8.6%–106.3%). This preliminary study revealed the effectiveness of zanubrutinib-containing regimens in DLBLC, especially CNS-involved cases, for the first time. The excellent BBB penetration of zanubrutinib supports its further investigation for the treatment of CNS lymphoma.

## 1 Introduction

B-cell receptor (BCR) signaling is essential for normal B-cell proliferation, differentiation, survival, and function. Aberrant BCR signaling pathway activation is consequently observed in various B-cell malignancies (1). Therefore, blocking BCR signaling is an attractive strategy for therapeutic intervention in B-cell malignancies. Bruton’s tyrosine kinase (BTK) is a key effector of BCR signaling. BTK inhibitors including ibrutinib, zanubrutinib, and acalabrutinib show significant clinical effects in chronic lymphocytic leukemia (CLL), Waldenström macroglobulinemia (WM), and mantle cell lymphoma (MCL). The first-generation BTK inhibitor ibrutinib shows significant antitumor activity in relapsed/refractory activated B cell-like (ABC) diffuse large B-cell lymphoma (DLBCL) (2). Very recently, after analysis of biopsies from Phoenix trial, Wilson. et al. reported that the 3-year event-free survival of younger patients treated with ibrutinib plus R-CHOP was 100% in the MCD subtypes, which provide solid evidence of the efficacy of ibrutinib in DLBCL (3) .

Central nervous system lymphoma (CNSL) remains a great clinical challenge; relapse eventually occurs in over 60% of primary CNSL cases, and the median overall survival period is less than 12 months for secondary CNSL (4, 5). The unmet need for effective biological therapies stems from the impermeability of the blood–brain barrier (BBB). The BBB restricts entry to the brain due to its very low paracellular permeability. Only lipophilic drugs can easily cross the BBB by passive diffusion, while some can permeate the BBB *via* potent influx transporters (6). Drug efficacy in CNSL requires the drug to pass through the BBB and distribute in the brain. Methorexate (MTX) based protocols are widely used, such as MA (methotrexate, cytarabine), MATRiX (MTX, cytarabine, thiotepa, rituximab), MVP (MTX, vincristine, procarbazine), however, the complete remission rate was less than 60% (7, 8). Adding small molecular drugs with good penetration of brain blood-barrier (BBB) may induce higher response rate, such as lenalidomide (NCT04120350). Secondly, the optimal prophylaxis strategy is undetermined. Intrathecal or intravenous MTX has been used for a longtime, but the efficacy was disappointed, the CNS relapsing rate was >10% in the high risk group after high-dose MTX (9, 10). In view of the above-mentioned facts, we need a novel medication with good BBB penetration to overcome difficulties in CNLS treatment.

BTK inhibitors may be effective for primary central nervous system lymphoma (PCNSL). Various case reports have demonstrated that ibrutinib crosses the BBB, leading to complete remission (11–14). Analytical methods have been reported for quantifying the active metabolites of ibrutinib in animal models along with human plasma and cerebrospinal fluid (CSF) (15–18). Tirabrutinib, another BTK inhibitor, has also shown good efficacy in patients with relapsed/refractory PCNSL and was approved on March 25, 2020 in Japan (19).

Zanubrutinib (BRUKINSA™, Beigene Corporation; also known as BGB-3111) is an irreversible next-generation BTK inhibitor that was approved for the treatment of recurrent or refractory CLL, MCL, and WM (20–22). Zanubrutinib was designed with favorable pharmacokinetic and pharmacodynamic (PD) features to maximize BTK occupancy and minimize off-target inhibition (23).

However, there is no clinical evidence of zanubrutinib in DLBCL with CNS involvement. Therefore, we reviewed the clinical profiles of several aggressive B-cell lymphoma cases treated with zanubrutinib to investigate the effectiveness and safety of zanubrutinib. We hypothesized that zanubrutinib effectively permeates the BBB and is effective for the treatment of primary and secondary CNSL. To the best of our knowledge, no method has been specifically developed for the quantification of zanubrutinib in the CSF. Thus, we developed a sensitive and rapid liquid chromatography–tandem mass spectrometry (LC-MS/MS) method for the quantification of zanubrutinib in plasma and CSF, evaluated the distribution of zanubrutinib in CSF, and preliminarily assessed the safety and effectiveness of zanubrutinib against DLBCL.

## 2 Materials And Methods

### 2.1 Patients

Patients with treatment-naive and relapsed/refractory DLBCL were enrolled in this study. The inclusion criteria were as follows: age > 18 years; histopathologically confirmed DLBCL (including DLBCL-NOS, PCNSL, and IVLBCL); assessable lesions by computed tomography or magnetic resonance imaging; and treatment with zanubrutinib-containing regimens. The exclusion criteria were as follows: severe disease complications or serious comorbidities; active infection; uncontrolled bleeding events; and pregnancy or lactation in women.

This study was conducted in accordance with the ethics principles of the Declaration of Helsinki, and the study methods were approved by the Institutional Review Board of Peking Union Medical College Hospital. Informed consent was obtained from all patients.

### 2.2 Treatment

The dose of zanubrutinib was fixed at 160 mg twice per day in all patients. Treatment duration and the combined drugs/regimens were decided by the clinicians.

Four regimens were evaluated in this study: Regimen 1, zanubrutinib + rituximab in primary vitreoretinal lymphoma (PVRL) (zanubrutinib at 160 mg twice per day until disease progression or intolerance and rituximab at 375 mg/m^2^ every 21 days); Regimen 2, zanubrutinib combined with high-dose methotrexate with or without cytarabine (HD-MTX regimen/MA regimen) in CNSL (methotrexate at 3.5–5.0 g/m^2^ d1 and cytarabine at 2.0 g every 12 h on d2 and d3, every 21 days per cycle); Regimen 3, zanubrutinib combined with dexamethasone, ifosfamide, carboplatin, and etoposide (DICE) or dexamethasone, cytarabine, and cis-platinum (DHAP) in relapsed and refractory patients every 21 days per cycle as prescribed for the salvage chemotherapy of autologous stem cell transplantation (ASCT); and Regimen 4, zanubrutinib combined with rituximab, cyclophosphamide, epirubicin, vindesine, and prednisone (R-CHOP) for eight cycles as an initial treatment in IVLBCL.

### 2.3 Quantification of Zanubrutinib in Peripheral Blood and CSF Samples

#### 2.3.1 Plasma and CSF Sample Preparations

Zanubrutinib is absorbed rapidly with a median time to maximum concentration of 2 h. Blood and CSF samples were collected at 2 or 3 h after the oral administration of zanubrutinib at 160 mg. Zanubrutinib administration was performed for more than 7 d. Additional plasma and CSF specimens for trough concentration testing were collected just before zanubrutinib administration (i.e., 12 h after the last zanubrutinib dose). Biological samples were stored at −20°C for a maximum of one month before analysis.

#### 2.3.2 Materials, Instruments, and Procedures

Zanubrutinib in CSF and sodium heparin plasma samples was quantified by LC-MS/MS. Zanubrutinib standard was obtained from Beigene (Beijing, China). Methanol, methanoic acid, and acetonitrile (high-performance liquid chromatography gradient grade) were purchased from Fisher Scientific, USA. Ultrapure water was obtained using a Milli-Q purification system (Millipore, MA, USA). Control plasma and CSF samples for standards and quality control were obtained from patients treated in the department of hematology of PUMCH. The detailed LC-MS/MS procedure is described in the **Supplementary Material**.

### 2.4 Clinical Outcomes and Adverse Events (AEs)

The clinical response was assessed based on the Lugano 2014 Criteria for DBLCL and 2005 IELSG Criteria for PCNSL (24, 25). AEs were recorded and assessed according to the Common Terminology Criteria for Adverse Events (CTCAE) v4.0.

### 2.5 Statistical Analysis

Continuous variables were expressed as median and range and compared by Mann–Whitney U-test. Categorical variables were reported as percentages and compared by chi-squared test. The duration of response (DOR) was determined using the Kaplan–Meier method. Statistical analyses were performed with SPSS 25.0 (IBM, NY, USA) or Prism 7.0 (GraphPad, CA, USA). *P* < 0.05 was considered statistically significant.

## 3 Results

### 3.1 Clinical Characteristics and Treatment

A total of 13 patients were included from August to December 2020. The median age was 53 (range, 39–69) years, and four patients were male. The histological subtypes were PCNSL (*n* = 8), DLBCL-NOS (*n* = 2), PTL (*n* = 1), and IVLBCL (*n* = 2); all eight PCNSL cases were PVRL with or without brain involvement. The median IELSG score of the PCNSL cases was 0 (0–1). The median IPI (International Prognosis Index) score of the five systemic DLBCL cases was 4 (0–4), and four out of those five cases were Ann Arbor IV stage. A total of seven cases were relapsed, with a median of two (range, 1–3) prior treatments; six patients were newly diagnosed with primary vitreoretinal lymphoma and IVLBCL. A total of seven patients had CNS involvement; of these, four cases showed PRVL relapse with intracerebral lesions, and three had DLBCL with systemic and intracranial lesions. The detailed clinical and histopathological characteristics are shown in **Table 1**.

**Table 1:** Patient demographics and baseline characteristics.

ID	Age	Sex	Diagnosis	Comorbidity	Status	Lesion location @diagnosis	Previous treatments	Lesion location @relapsing	Regimen	Best response/DOR
1	69	M	PCNSL	HTN	TN	eyes	NA	NA	R+Zan	CR/11m
2	53	F	PCNSL	Ovarian tumor	TN	eyes	NA	NA	R+Zan	CR/9m
3	44	F	PCNSL	HCV	TN	eyes	NA	NA	R+Zan	CR/9m
4	52	F	PCNSL		TN	eyes	NA	NA	R+Zan	CR/9m
5	50	F	PCNSL	breast cancer, paraganglioma	relapsed	eyes	rituximab/Lenalidomide×6,R-MTX×2, etoposide×2	left frontal lobe, cerebellum	Zan+MA	CR/14m
6	62	F	PCNSL	hypertension	relapsed	eyes	rituximab/lenalidomide×6	right frontal lobe	Zan+R+HD-MTX	CR/13m
7	59	F	PCNSL	HBV	relapsed	eyes	Rituximab+lenalidomide×6,	bilateral basal ganglia	Zan+HD-MTX	SD/NA
8	52	F	PCNSL		relapsed	eyes	rituximab/Lenalidomide×6,	bilateral frontal lobes, callosum	Zan+R+HD-MTX	CR/13m
9	63	M	PTL		refractory	testis, epididymis	R-EPOCH/R-MA	testis, epididymis	Zan+R-DICE	CR/11m
10	39	F	DLBCL, NOS	HBV	relapsed	bone marrow, lung, uterus	R-MTX-CHOP, ASCT,TEDDI-R	right frontal lobe	Zan+MA	PD/NA
11	60	M	DLBCL, NOS		relapsed	cervical lymph nodes		muscles of right thigh	Zan+R-DHAP	CR/13m
12	65	M	IVBCL	diabetes mellitus	TN	lung, bone marrow, liver	NA	NA	Zan+R-CHOP	CR/12m
13	53	F	IVBCL	hypothyroidism	TN	CNS, skin, lung	R-CHOP-MTX×8	NA	Zan	PR/13m

COO, cell of origin; CNS, central nerves system; CR, complete remission; CHOP, cyclophosphamide, epirubicin, vindesine, prednisone; DICE, dexamethasone, ifosfamide, carboplatin and etoposide; DHAP, dexamethasone, cytarabine and cis-platinum; DOR, duration of response; EPOCH, etoposide, prednisone, vindesine, cyclophosphamide, epirubicin; F, female; GCB, germinal center B cell; HD, high dose; HBV, hepatitis B virus; HCV, hepatitis C virus; IVLBCL, intravascular large B cell lymphoma; MA, methotrexate, cytarabine; M, male; MTX, methotrexate, MT, maintenance; PCNSL, primary central nervous system lymphoma; PD, progressed disease; PR, partial remission; PTL, primary testis lymphoma; R, rituximab; TN, treat naïve; NA, not applicable; Zan, zanubrutinib.

**Figure 1:**
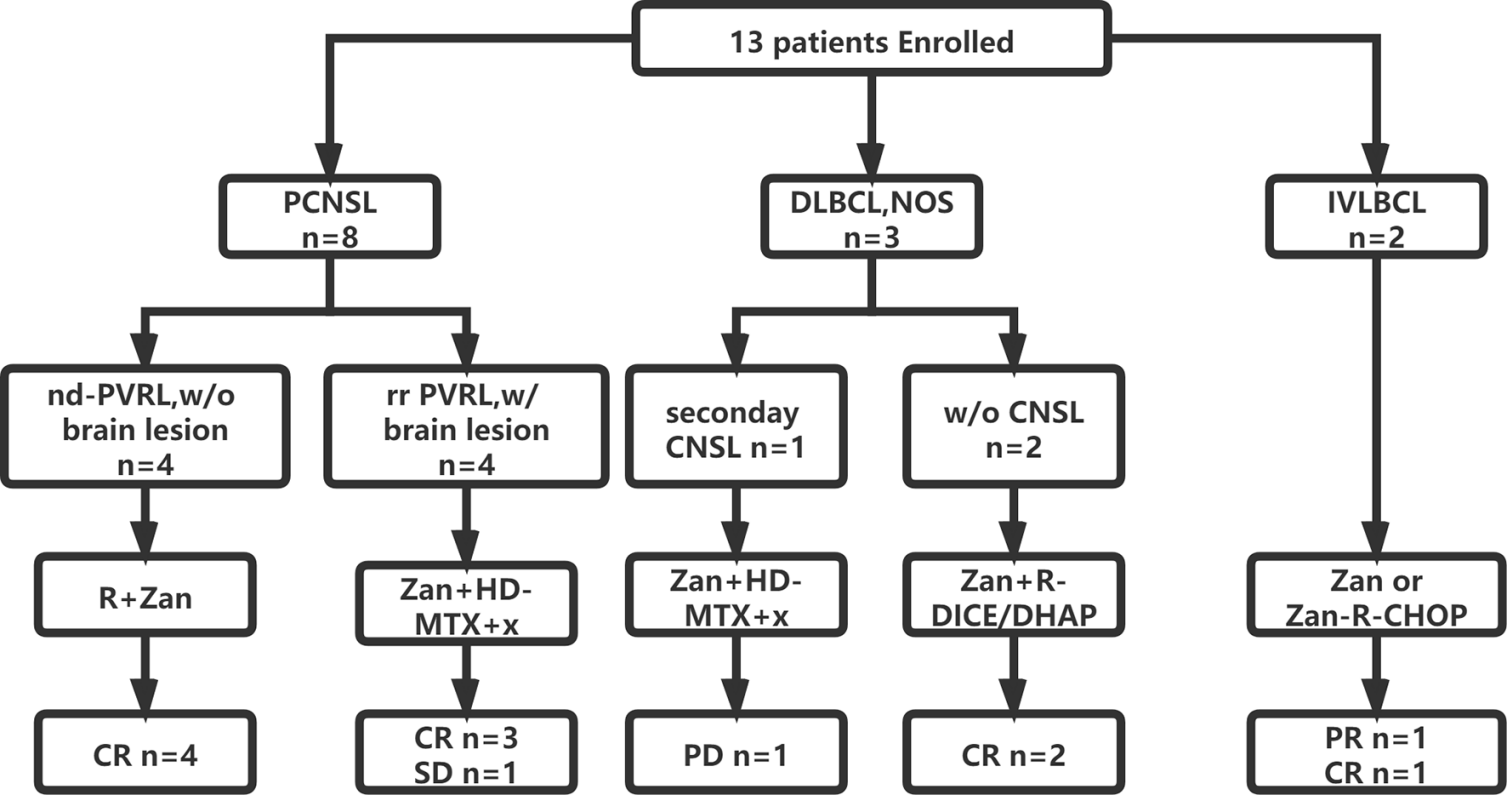
Flowchart of patient enrollment, treatment and outcome. CNS, central nerves system; CR, complete remission; CHOP, cyclophosphamide, epirubicin, vindesine, prednisone; DICE, dexamethasone, ifosfamide, carboplatin and etoposide; DHAP, dexamethasone, cytarabine and cis-platinum; HD, high dose; IVLBCL, intravascular large B cell lymphoma; MTX, methotrexate; nd, newly-diagnosed; R, rituximab; PCNSL, primary central nervous system lymphoma; PVRL, primary vitreoretinal lymphoma; PR, partial remission; PD, progressed disease; w/, with; w/o, without; Zan, zanubrutinib.

A total of four PVRL patients without CNS involvement were treated with zanubrutinib and rituximab as a first-line treatment. Zanubrutinib and high-dose (HD) methotrexate (MTX)-based regimens were applied in five patients with isolated CNS involvement; these regimens included MTX combined with rituximab and/or cytarabine (HD-MTX, *n* = 1; R-HD-MTX, n = 2; R-MA, *n* = 2). Two patients with relapsed/refractory DLBCL received rituximab and zanubrutinib combined with DICE or DHAP as salvage regimens. Two patients with IVLBCL had systemic CNS involvement at diagnosis; one was treated with zanubrutinib combined with R-CHOP as a first-line therapy, while the other received R-CHOP-MTX for eight cycles and achieved partial response (PR). The patient achieving PR was further treated with zanubrutinib as a maintenance therapy (**Figure 1**).

### 3.2 Clinical Outcomes

All five treatment-naïve patients (one IVBLC and four PRVL patients) achieved complete response (CR) after six to eight cycles of zanubrutinib-containing first-line regimens. The two relapsed/refractory DLBCL cases without CNSL involvement achieved CR and received ASCT as consolidation.

**Figure 2:**
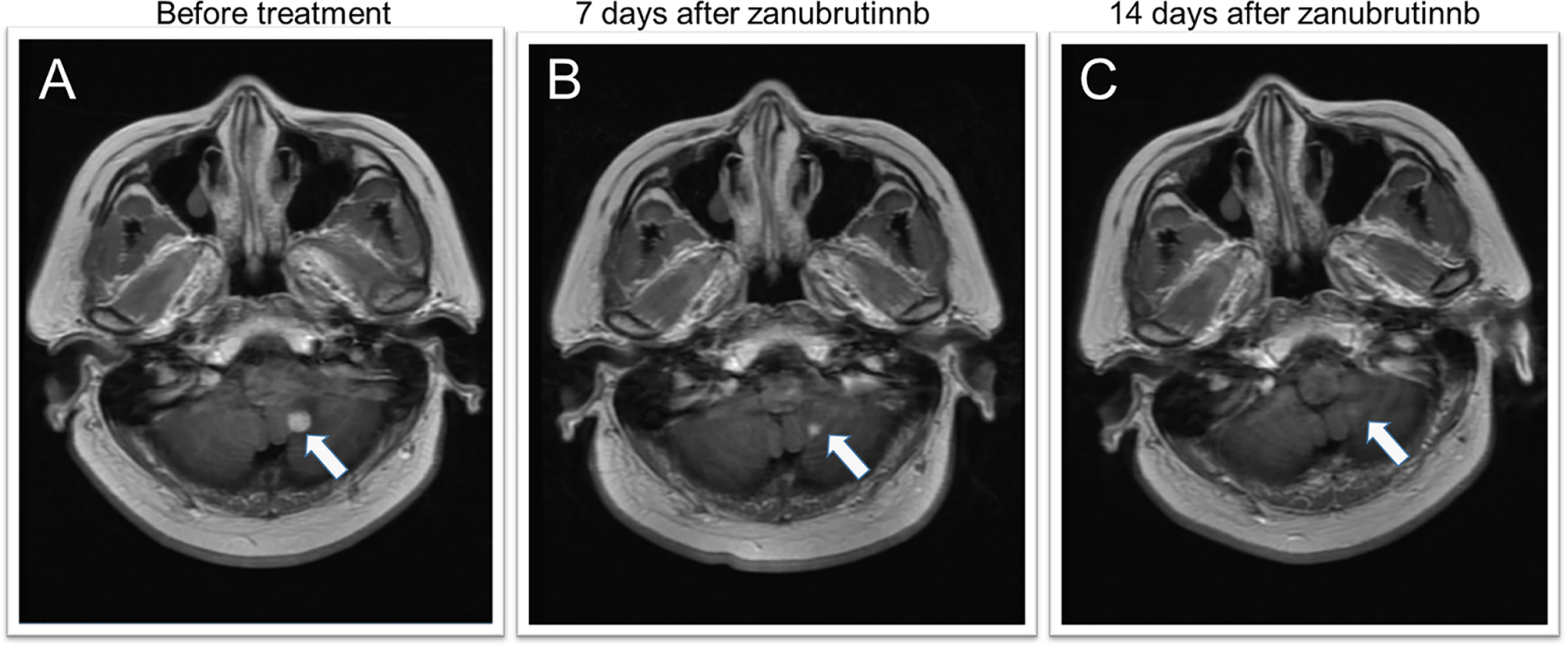
MRI features of a secondary CNSL case during zanubrutinib monotherapy. **(A)** MRI depicts a homogeneously contrast-enhanced mass in the left cerebellum before zanubrutinib administration. **(B, C)** 7 and 14 days after zanubrutinib, the mass shrank and disappeared.

**Figure 3:**
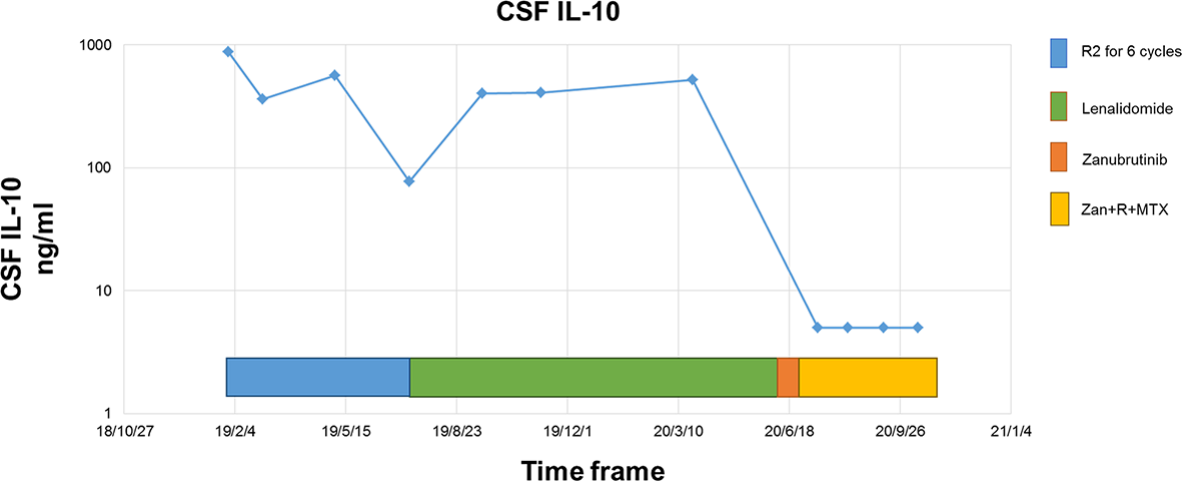
Dynamic change of CSF IL-10 concentrations during the whole treatment.

The response rate for the five relapsed CNSL patients was 60% (3/5), including three patients with CR, one with stable disease (SD), and one with disease progression. Of these five patients, two first received zanubrutinib monotherapy for over seven days as a lead-in therapy before combination chemotherapy, and a rapid and dramatic response was observed in both of these patients. Case 5 was a 50-year-old woman diagnosed with PRVL who relapsed in bilateral eyes and the frontal lobe in sequence after first-line treatment with rituximab and lenalidomide. Short durations of complete remission (four and three months, respectively) were achieved after the administration of rituximab combined with HD MTX and HD etoposide as salvage treatments. At the third relapse, she was diagnosed with bladder paraganglioma that required surgery. Zanubrutinib was administered only around the operative period; consequently, the lesion in the cerebellum shrank and disappeared within three weeks (**Figure 2**). Case 6 was a 62-year-old female with PRVL who was treated with rituximab and lenalidomide as a frontline treatment followed by lenalidomide as a maintenance therapy. Although the patient achieved CR according to IELSG Criteria 2005, the amount of IL-10 in the CSF remained around 400 ng/ml (normal range, < 5 ng/ml). The CSF IL-10 concentration is strongly associated with the clinical response and/or progression (26, 27). After the disease relapsed 18 months later, the patient underwent zanubrutinib monotherapy for 7 d before systemic intravenous MTX administration. The CSF IL-10 concentration was dramatically decreased by the zanubrutinib monotherapy, which was related to the control of CNS lymphoma and demonstrates the significant anti-tumor activity of zanubrutinib (**Figure 3**).

The overall response rate (ORR) of the entire population was 84.5% (11/13), and the CR rate was 76.9% (10/13). After a median follow-up of 3.6 (6–14) months, the DOR was 12 (9–14) months. Only one patient died from disease progression.

Due to the heterogeneity of the zanubrutinib-based regimens, the categories and severity of AEs varied greatly among the different regimens. The median treatment cycle number was four (range, 2–8). The treatment was discontinued early because of disease progression in only two cases, and the remaining patients completed the planned cycles (*n* = 4) or were still under treatment (*n* = 7). The most common AEs (> 10%) were neutropenia, thrombocytopenia, infection, nausea, vomiting, abnormal alanine transaminase, and mucositis; no bleeding or cardiac events occurred during treatment (**Table 2**).

**Table 2:** Adverse reactions of zanubrutinib-containing regimens.

AE	Severity	Zan+R (*n* = 5)	Zan+MTX ± R (*n* = 3)	Zan+MA (*n* = 2)	Zan+CHOP (*n* = 1)	Zan+DICE/DHAP (*n* = 2)	Total (*n* = 13)
neutropenia	**any grade**	1	3	2	1	2	9
	**3-4 grade**	0	1	2	1	1	5
Thrombocytopenia	**any grade**	0	1	2	0	1	4
	**3-4 grade**	0	0	1	0	0	1
infection	**any grade**	1	0	2	0	1	4
	**3-4 grade**	0	0	1	0	0	1
Febrile neutropenia	**any grade**	0	0	1	0	1	2
anemia	**any grade**	0	1	2	1	2	6
	**3-4 grade**	0	0	2	0	0	2
Vomit/nausea	**any grade**	1	3	2	1	2	9
	**3-4 grade**	0	0	0	0	1	1
Abnormal liver function	**any grade**	1	1	1	0	1	4
	**3-4 grade**	0	0	0	0	0	0
Renal injury	**any grade**	0	0	0	0	0	0
	**3-4 grade**	0	0	0	0	1	1
Mucositis	**any grade**	0	1	2	0	0	3
	**3-4 grade**	0	0	1	0	0	1
Fatigue	**any grade**	3	2	2	1	1	9
	**3-4 grade**	0	0	1	0	1	2
Bleeding event	**any grade**	0	0	0	0	0	0
Diarrhea	**any grade**	0	0	0	0	0	0
Arrhythmia	**any grade**	0	0	0	0	0	0
Diarrhea	**any grade**	0	0	0	0	0	0

CHOP, cyclophosphamide, epirubicin, vindesine, prednisone; DICE, dexamethasone, ifosfamide, carboplatin and etoposide; DHAP, dexamethasone, cytarabine and cis-platinum. R, rituximab; Zan, zanubrutinib.

### 3.3 CSF Distribution of Zanubrutinib

A total of 23 time-matched plasma-CSF sample pairs were collected from the 13 patients. Due to the rapid absorption and distribution of zanubrutinib, the time to maximum concentration is approximately 2 h. Therefore, the sampling time was selected as 2–3 h after zanubrutinib administration.

**Figure 4:**
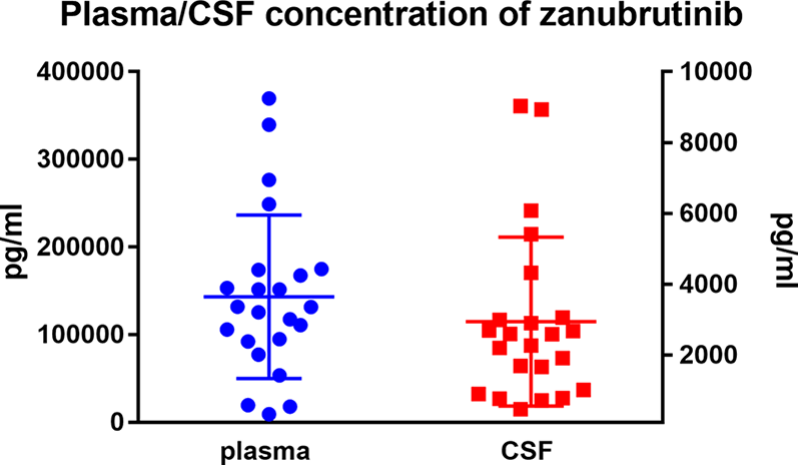
Zanubrutinib concentrations of plasma and CSF.

The mean plasma concentration of zanubrutinib was 143190.6 ± 93302.7 pg/ml (range, 9281.0–369380.0 pg/ml) at the peak time point median CSF concentration was 2941.1 ± 2382.01 pg/ml (range, 466–9032.0 pg/ml) (**Figure 4**). The median CSF/plasma ratio was 2.39% ± 1.71% (range, 0.61%–5.83%), indicating a low BBB penetration for zanubrutinib. However, considering the high protein binding rate of 94%, the corrected CSF/plasma ratio was 42.7% ± 27.7% (range, 8.6%–106.3%). The increase in this corrected CSF/plasma ratio demonstrates that free zanubrutinib was successfully transported from the blood into the CSF. The peak CSF concentrations of zanubrutinib in 95.7% (22/23) of the samples were above the enzymatic IC50 values (ABC DLBCL lines *in vitro*, IC50 = 118pg/ml, 0.4nM; OCI-Ly-10, IC50 = 707.34 pg/ml 1.5nM).

A comparison of the zanubrutinib CSF/plasma ratios of different patients showed no significant effects of sex, DLBCL subtype, CNS involvement, or the co-administration of BBB-penetrating drugs (*P* > 0.1).

We compared the plasma and CSF concentrations of zanubrutinib at different time points (2 vs. 3 h after administration). While the plasma and CSF concentrations of zanubrutinib tended to be higher in the samples collected at 2 h after administration, the penetration ratios were similar between the two time points (**Table 3**).

**Table 3:** Zanubrutinib concentrations in plasma and CSF.

	Interval between administration and sampling	Sample	Mean concentration(pg/ml)	*P*-value
Plasma	2 h	10	182909.3 ± 91077.1	0.193
	3 h	13	125813.7 ± 91605.7	
	entire series	23	143190.6 ± 93302.7	
CSF	2 h	10	4117.0 ± 2864.3	0.192
	3 h	13	2426.6 ± 2028.0	
	entire series	23	2941.1 ± 2382.01	
Corrected CSF/plasma ratio (%)	2 h	10	41.2 ± 32.5	0.869
	3 h	13	43.3 ± 26.5	
	entire series	23	42.7 ± 27.7	

**Figure 5:**
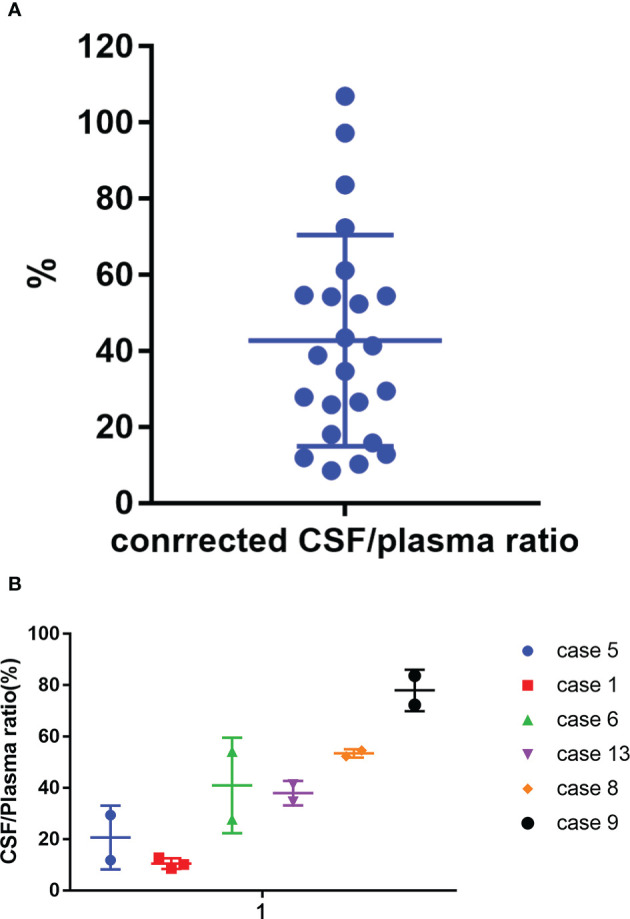
Corrected CSF/plasma ratios for zanubrutinib. **(A)** 23 paired samples of Corrected CSF/plasma ratio by protein binding rate. **(B)** Corrected CSF/plasma ratios for zanubrutinib in different patients.

Meanwhile, we tested reduplicated samples collected from six patients at the same time point but in different cycles. We observed huge variation in the CSF/plasma ratio among these patients, with values ranging from 10.5% to 78.1%. However, the consistency was acceptable between cycles in the same patient (**Figure 5** and **Table S1**).

We also tested the trough concentrations of zanubrutinib in two patients (case 1 and case 3) to evaluate if the CSF concentrations of zanubrutinib remained roughly above the IC50 value. The CSF concentrations were 267 and 5022 pg/ml in case 1 and case 3, respectively, and case 3 showed a value below the IC50 value.

## 4 Discussion

As mentioned above, there are great unmet needs in CNSL treatment and prophylaxis, BTK inhibitors (ibrutinib and tirabrutinib) show efficacy in CNSL. To the best of our knowledge, this is the first study to demonstrate the effectiveness of zanubrutinib-containing regimens in different subtypes of DLBCL, particularly CNS lymphoma. The high BBB penetration of zanubrutinib was confirmed by LC-MS/MS, which supported the use of zanubrutinib in CNSL.

This case study consisted of 13 DLBCL patients treated with zanubrutinib-containing regimens with an ORR of 84.6%. Two relapsed cases with PCNSL received zanubrutinib alone as a lead-in treatment at the beginning of salvage therapy, and both achieved significant disease control within 14 d. Lionakis et a. reported a similar phenomenon for ibrutinib in 2017; they conducted a 14-d therapy with ibrutinib before applying TEDDI-R as a combination regimen, and 94% of patients showed disease improvement after the ibrutinib monotherapy (28). This promising result indicates that zanubrutinib can induce rapid remission in CNSL patients. Thus, zanubrutinib could be a good option for these patients.

We also evaluated the safety of zanubrutinib-containing regimens in DLBCL. The tolerability of zanubrutinib was favorable in this study. Significant toxicity was indicated by hematological events; grade 3–4 neutropenia and thrombocytopenia occurred in 54.5% and 11.1% of cases, respectively. All severe AEs occurred for intensive salvage regimens, including HD cytarabine, DICE, and DHAP. In a phase I study of ibrutinib combined with R-ICE for the treatment of transplant-eligible r/r DLBCL, no dose-limiting toxicities were observed, even with ibrutinib at 840 mg daily (29). In our study, both cases of relapsed DLBCL achieved CR with the zanubrutinib-containing regimens, and no unexpected serious AEs were observed. Meanwhile, no unique AEs related to BTK inhibitors (e.g., bleeding events, atrial fibrillation, and aspergillosis) were observed. The low incidence of BTK inhibitor-specific AEs was attributed to the limited number of cases in this study; the low off-target inhibitory activity of zanubrutinib may also have contributed (22).

However, zanubrutinib has not been approved in DLBCL/PCNSL. We applied zanubrutinib in DLBCL/PCNSL based on the following scientific and ethical considerations. First, evidence regarding the efficacy of zanubrutinib in DLBCL emerged in 2017. At the 2017 American Society of Hematology meeting, Tam et al. reported that among 75 patients with non-Hodgkin lymphoma (including 23 with DLBCL) who were treated with zanubrutinib, 14 achieved remission (30). In 2021, Yang et al. reported the application of zanubrutinib in relapsed non-GCB (germinal center) DLBCL in a multicenter, single-arm, phase 2 study involving 41 patients (31). After a median follow-up time of 6.8 months, the ORR was 29.3%, and the complete response rate was 17.1%. AEs leading to treatment discontinuation were reported in four patients. Second, the efficacy and safety of zanubrutinib as a monotherapy or combined with R-CHOP were confirmed by data from two trials conducted at our center: Intravitreal MTX and ZR Regimen in Newly Diagnosed PVRL, NCT04899453 (Regimen 1); and Zanubrutinib Combined With R-CHOP in Newly-diagnosed Intravascular Large B-cell Lymphoma, NCT04899570 (Regimen 4). These results give us confidence to expand the application of zanubrutinib in DLBCL. Third, the best salvage regimen was undermined in relapsed DLBCL and PCNSL, and clinical trials were preferred. The patients from Regimen 2 and Regimen 3 were relapsed/refractory (r/r) DLBCL with/without CNS involved; these patients were treated with a zanubrutinib-containing regimen after fully understanding the benefits and risks and signing the informed consent forms. All activities were approved by the Institutional Review Board of our institution.

In this study, we analyzed the distribution of zanubrutinib in CSF for the first time. Some issues should be clarified. First, zanubrutinib maybe a suitable candidate for primary and secondary CNSL treatment with outstanding BBB permeability. The median CSF/plasma ratio of zanubrutinib was around 2%; after adjusting for the amount of protein-bound drug, the CSF/plasma ratio increased to nearly 40%. This significant change demonstrates that zanubrutinib penetrated the BBB in its free form. Several studies have demonstrated the high brain distribution level of ibrutinib in animal models and human biological specimens (15, 16, 18). In 2016, Soussain et al. confirmed that ibrutinib can penetrate in the BBB, with CSF concentrations above the IC50 level consistent with clinical response (14). In 2017, Lionakis et al. confirmed the high efficacy of an ibrutinib-containing regimen (TEDDi-R) in CNS lymphomas and evaluated the pharmacokinetics of ibrutinib in plasma and CSF samples (28). After adjusting for a protein binding of 97.3%, the authors found a CSF/plasma ratio of 28.7% (23.2%–446.6%), and the median time that the CSF concentration remained above the enzymatic IC50 level was 4 h (0–24 h) at a dose of 840 mg. In 2018, Goldwirt et al. developed a Swiss mouse model to evaluate the distribution of ibrutinib in the brain and found that ibrutinib crossed the BBB and was highly distributed in the brain tissue (18). In 2020, Narita et al. reported the CSF concentration of tirabrutinib in PCNSL patients, the trough concentrations at 320 mg and 480 mg were 2.19 ± 0.476 and 14.0 ± 8.92) ng/mL, respectively (19). We compared the plasma and CSF concentrations of different BTK inbihotors (see **Table 4**), zanubrutinib had higher CSF concentration than ibrutinib and low dose tirabrutinib.

**Table 4:** Plasma and CSF concentrations of different BTK inhibitors.

	Ibrutinib			Tirabrutinib		Zanubrutinib
Dose(mg)	560mg qd	700mg qd	840mg qd	320mg qd	480mg qd	160mg bid
Mean Plasma(ng/ml)	53.7	217.4	875.6	16.3	77.0	143.2
Mean CSF (ng/ml)	0.62	0.87	0.59	2.19	14.0	2.94

Second, according to the package insert, the median time to maximum concentration of zanubrutinib is 2 h, resulting in a mean half-life of approximately 2–4 h. We then compared the CSF and plasma concentrations of zanubrutinib at different time points. The plasma concentrations of zanubrutinib were higher at 2 h after administration compared to at 4 h after administration; a similar trend was found in the CSF concentrations. This suggests similar dynamics in the CSF and plasma concentrations of zanubrutinib, with rapid and efficient BBB crossing. This distribution behavior of zanubrutinib is similar to that of ibrutinib (18).

Another issue is the absolute zanubrutinib concentration in the CSF. The difference between the highest and lowest zanubrutinib concentrations was almost 200 times, with values ranging from 466 to 8930 pg/ml. Zanubrutinib showed antitumor activity in the pg range in two ABC DLBCL lines (TMD8, IC50 = 118 pg/ml; OCI-Ly-10, IC50 = 707.34 pg/ml) (32). While a zanubrutinib concentration below the IC50 value was found in one CSF sample, the mean concentration of zanubrutinib in the CSF was much higher than the IC50 value. The lowest concentration was detected in case 9, a female patient who showed a rapid response to zanubrutinib monotherapy. This highlighted the potential effect of zanubrutinib/ibrutinib for prevention of CNS relapse in DLBCL.

This preliminary study had several limitations. First, the present results are based on a limited number of patients, and the DLBLC and treatment subtypes were heterogeneous. This may generate selection bias. Second, detailed pharmacokinetic data were not collected for zanubrutinib. No Ommaya reservoirs were placed in the patients, and CSF samples were collected by lumbar puncture, allowing for only one sample during each 24-h treatment cycle. Without dynamic monitoring, comprehensive information about the CSF distribution, including how long the CSF concentration stayed above the IC50 value and the area under the curve of CSF, could not be obtained. Finally, prolonged effectiveness was not evaluated because of the limited observation period.

## 5 Conclusions

The high response rate and good tolerance observed in this study suggest that zanubrutinib-containing regimens are a promising treatment option for DLBCL, especially CNSL. Zanubrutinib could effectively cross the BBB. The results provide preliminarily evidence for the application of zanubrutinib in CNS lymphoma.

## Data Availability Statement

The original data presented in this paper are included in the article/**Supplementary Material**. Further inquiries can be directed to the corresponding author.

## Ethics Statement

The studies involving human participants were reviewed and approved by Institutional Review Board of Peking Union Medical College Hospital. The patients/participants provided their written informed consent to participate in this study.

## Author Contributions

YZ, YL, and ZZ performed the research. YZ and WZ designed the study. YZ, WW, DQZ, and CW took care of the patients. YZ analyzed the data. YZ wrote the paper. DBZ supervised the study and revised the paper. All authors read and revised the manuscript, and all authors have approved the submitted version.

## Funding

This study was funded by the CAMS Innovation Fund for Medical Sciences (CIFMS) [2021-I2M-C&T-B-005] and CAMS Innovation Fund for Medical Sciences (CIFMS) [2019-I2M2-009].

## Conflict of Interest

The authors declare that the research was conducted in the absence of any commercial or financial relationships that could be construed as a potential conflict of interest.

## Publisher’s Note

All claims expressed in this article are solely those of the authors and do not necessarily represent those of their affiliated organizations, or those of the publisher, the editors and the reviewers. Any product that may be evaluated in this article, or claim that may be made by its manufacturer, is not guaranteed or endorsed by the publisher.
