# Supplementary material for: Preliminary Evaluation of Zanubrutinib-Containing Regimens in DLBCL and the Cerebrospinal Fluid Distribution of Zanubrutinib: A 13-Case Series
Source: Front Oncol. 2021 Dec 24;11:760405. doi: 10.3389/fonc.2021.760405 (PMC8739956; doi:10.3389/fonc.2021.760405)
Supplement: Supplementary file 1 [file Table_1.docx]

# Supplementary material

**Table S1. Zanubrutinib concentrations in plasma and CSF and the mean corrected CSF/plasma ratio**

| Patient | Duration of zanubrutinib administration (d) | Interval between zanubrutinib administration and CSF/plasma sampling (h) | Concentration in plasma (pg/mL) | Concentration in CSF (pg/mL) | Corrected CSF/plasma ratio (%) | Mean corrected CSF/plasma ratio (%) |
| --- | --- | --- | --- | --- | --- | --- |
| Case 8 | 49 | 2 | 125509 | 896.4 | 11.90 | 20.70 |
| Case 8 | 90 | 2 | 151559 | 2682 | 29.49 |  |
| Case 12 | 30 | 3 | 369380 | 2264 | 10.22 | 10.54 |
| Case 12 | 72 | 3 | 131343 | 1009 | 12.80 |  |
| Case 12 | 96 | 3 | 151575 | 782 | 8.60 |  |
| Case 1 | 121 | 3 | 173748 | 2900 | 27.81 | 41.00 |
| Case 1 | 61 | 3 | 92104 | 2995 | 54.20 |  |
| Case 4 | 14 | 3 | 105917 | 2200 | 34.62 | 38.00 |
| Case 4 | 42 | 3 | 77024 | 1913 | 41.39 |  |
| Case 5 | 92 | 3 | 53606 | 1684 | 52.36 | 53.50 |
| Case 5 | 18 | 3 | 131792 | 4321 | 54.64 |  |
| Case 6 | 92 | 3 | 9281 | 466 | 83.68 | 78.01 |
| Case 6 | 8 | 3 | 17669 | 766.9 | 72.34 |  |

**Supplementary Material: Liquid chromatography–tandem mass spectrometry (LC-MS/MS) procedure**

Zanubrutinib solutions were prepared at a concentration of 1 mg/ml in methanol and further diluted with methanol to obtain working solutions at concentrations of 100–1,000,000 pg/ml. Biological samples (50 µl) were mixed with 10 µl of methanol and 100 µl of acetonitrile. The mixed samples were centrifuged at 2,500 rpm for 3 min at room temperature and at 17,500 rpm for 10 min at 2°C–8°C. The supernatant (120 μl) was then injected into the chromatography system at room temperature.

The analytes were analyzed by reverse-phase chromatography using an UPLC XevoTQ-XS system (Waters Corp., Milford, MA, USA) equipped with an ACQUUITY UPLC®BEH C18 column (2.1×50 mm, 1.7 μm; Waters Corp.). The mobile phase consisted of 0.1% formic acid in acetonitrile and ultrapure water. A mass spectrometer was used in positive mode with an electrospray ionization source. Multiple reaction monitoring mode was used to detect analytes and internal standards.

To create a stock solution of zanubrutinib with a concentration of 1 mg/ml, zanubrutinib (4.55 mg) was added to methanol (4.55 ml). The standard working solution and quality control standard solution of zanubrutinib were then obtained by diluting the stock solution to 1 μg/ml with methanol. To establish the standard curve, the blank control samples of plasma and CSF were added into 50-μl EP tubes followed by the addition of 10 μl standard working solution with different concentrations (see the table below).

| Standard curve samples | Human blank plasma. CSF volume (μl) | Standard curve | Concentration of standard curve (pg/ml) | Volume of standard curve sample (μl) | Concentration of volume of standard curve (pg/ml) |
| --- | --- | --- | --- | --- | --- |
| CAL1 | 50 | Zanubrutinib-CWS1 | 100 | 10 | 20 |
| CAL2 | 50 | Zanubrutinib-CWS2 | 200 | 10 | 40 |
| CAL3 | 50 | Zanubrutinib-CWS3 | 400 | 10 | 80 |
| CAL4 | 50 | Zanubrutinib-CWS4 | 1600 | 10 | 320 |
| CAL5 | 50 | Zanubrutinib-CWS5 | 6400 | 10 | 1280 |
| CAL6 | 50 | Zanubrutinib-CWS6 | 12800 | 10 | 2560 |
| CAL7 | 50 | Zanubrutinib-CWS7 | 25600 | 10 | 5120 |
| CAL8 | 50 | Zanubrutinib-CWS8 | 32000 | 10 | 6400 |

The concentrations of analytes in CSF and plasma samples were obtained from the calibration curve constructed by plotting the peak area ratio of each analyte to the internal standard against the concentration. The analyte concentrations in the samples were calculated by linear regression.
